# Low von Willebrand factor—unraveling an enigma wrapped in a conundrum

**DOI:** 10.1016/j.jtha.2024.08.015

**Published:** 2024-09-10

**Authors:** James S. O’Donnell, Ross I. Baker, Ferdows Atiq

**Affiliations:** 1 Irish Centre for Vascular Biology, School of Pharmacy and Biomolecular Sciences, Royal College of Surgeons in Ireland, Dublin, Ireland; 2 National Coagulation Centre, St James’s Hospital, Dublin, Ireland; 3 Irish-Australian Blood Collaborative (IABC) Network; 4 Western Australia Centre for Thrombosis and Haemostasis, Perth Blood Institute, Murdoch University, Perth, Western Australia, Australia

**Keywords:** aging, diagnosis, Low VWF, type 1 VWD, von Willebrand disease

## Abstract

The 2021 ASH ISTH NHF WFH guidelines recommendation that patients with von Willebrand factor (VWF) levels of 30 to 50 IU/dL and an increased bleeding phenotype be categorized as type 1 von Willebrand disease (VWD) rather than Low VWF has proved controversial. However, in support of that decision, recent data have demonstrated that individuals with partial quantitative VWF deficiency exhibit an age-dependent evolving phenotype and confirmed that Low VWF represents a subgroup within heterogeneous type 1 VWD. Nonetheless, type 1 VWD heterogeneity continues to pose significant diagnostic challenges. In this Forum article, we address outstanding issues critical to preventing the inappropriate overdiagnosis of type 1 VWD while maximizing access to healthcare and minimizing diagnostic delays. In addition, we propose an algorithm for type 1 VWD diagnosis. This algorithm pays special attention to individuals with plasma VWF levels in the 30 to 50 IU/dL range who have no or minimal bleeding history and have not yet been exposed to significant hemostatic challenges.

## 1 | TYPE 1 VON WILLEBRAND DISEASE HETEROGENEITY AND THE LOW VON WILLEBRAND FACTOR CONCEPT

Type 1 von Willebrand disease (VWD) is caused by a partial quantitative reduction in plasma von Willebrand factor (VWF) levels and accounts for approximately 75% of VWD cases [1]. For many years, it has been clear that patients with type 1 VWD demonstrate marked clinical and laboratory heterogeneity. This includes interindividual differences in bleeding phenotype, wide variability in basal VWF levels, variable increases in plasma VWF levels in response to desmopressin, and fluctuations in VWF clearance rates [1–5]. In addition, previous studies have demonstrated interindividual differences in the pathogenic mechanisms underlying quantitative reductions in plasma VWF antigen (VWF:Ag) levels (reduced VWF biosynthesis and/or enhanced VWF clearance) [5–8]. Since VWF is a continuous trait, categorizing it into discrete diagnostic groups is challenging and does not fully represent the complexity underlying type 1 VWD biology. Nevertheless, in an effort to address the heterogeneous nature of partial quantitative VWD, previous international consensus guidelines from the National Heart, Lung, and Blood Institute in 2008 and the UK Haemophilia Doctors Organization in 2004 recommended that patients with partial quantitative VWD be subclassified into 2 distinct groups based on plasma VWF levels [9,10]. Thus, the guidelines proposed that patients with plasma VWF:Ag levels < 30 IU/dL and bleeding should be diagnosed with type 1 VWD. In contrast, patients with mild-to-moderate reductions in plasma VWF:Ag (in the range of 30–50 IU/dL) and bleeding should instead be diagnosed with Low VWF.

The concept of dividing partial quantitative VWD patients into type 1 (VWF:Ag < 30 IU/dL) and Low VWF (30–50 IU/dL range) subgroups was based on several lines of evidence [11]. For example, pathological *VWF* sequence variants and rare nonsynonymous *VWF* variants are both significantly more common in the cohort of patients with plasma VWF levels < 30 IU/dL [12–14]. Furthermore, VWD inheritance in families with plasma VWF levels < 30 IU/dL is commonly autosomal dominant in nature (albeit with variable penetrance) [7], whereas the inheritance pattern in families with plasma VWF levels in the 30 to 50 IU/dL range remains unclear [15]. Indeed, linkage analysis studies have demonstrated that the inheritance of mild-to-moderate reductions in plasma VWF:Ag levels is often not linked to the *VWF* gene [16]. These findings clearly raise questions about the value of genetic testing in patients with type 1 VWD, particularly in those with VWF levels in the 30 to 50 IU/dL range. From a clinical perspective, mucocutaneous bleeding in patients with plasma VWF levels < 30 IU/dL is common and correlates inversely with residual plasma VWF:Ag levels [7]. Conversely, current evidence suggests that most unselected individuals with mild-to-moderate reductions in plasma VWF:Ag levels (30–50 IU/dL) do not have any significant bleeding tendency [17,18]. Accordingly, it has been demonstrated that there is no benefit in routinely measuring VWF levels in unselected populations, such as prior to surgical interventions [18]. Instead, VWF levels should only be measured in individuals referred because of a personal bleeding phenotype or a positive family history of VWD. Furthermore, in the subgroup of patients with plasma VWF levels in the 30 to 50 IU/dL range who do have significant bleeding, the bleeding phenotype, as measured by a bleeding assessment tool score, does not correlate with residual VWF levels [19,20]. Collectively, these findings suggest that (i) additional modifier genetic loci contribute to the reduced plasma VWF levels seen in patients with Low VWF and (ii) other VWF-independent pathogenic mechanisms are important in determining mucocutaneous bleeding risk in patients with only mild-to-moderate reductions (30–50 IU/dL) in VWF.

## 2 | ASH ISTH NHF WFH GUIDELINES AND TYPE 1 VWD DIAGNOSIS

The American Society of Hematology (ASH), International Society on Thrombosis and Haemostasis (ISTH), National Hemophilia Foundation (NHF), World Federation of Hemophilia (WFH) guidelines published in 2021 addressed a number of specific questions prioritized following an initial international survey [21,22]. Based on a systematic review of the available evidence and in keeping with previous guidelines, the panel recommended that individuals with plasma VWF levels < 30 IU/dL should be diagnosed with type 1 VWD, irrespective of whether they had any previous bleeding [22]. Critically, however, in a significant change to previous guideline recommendations [9,10], the ASH ISTH NHF WFH guidelines also decided to remove the Low VWF category. Instead, the panel recommended that patients with abnormal bleeding and plasma levels in the 30 to 50 IU/dL range should also be diagnosed with type 1 VWD rather than “Low VWF.” The rationale for integrating the previous Low VWF and type 1 subgroup into a single group was that patients with VWF levels in the 30 to 50 IU/dL range and bleeding symptoms would find it easier to access clinical care in some jurisdictions (notably the United States) if they were registered with a formal diagnosis of type 1 VWD rather than Low VWF [22]. This enabling of access may be of particular importance in young women, in whom delays of up to 16 years from the onset of symptoms before VWD diagnosis have been reported [23]. Although the guidelines highlighted the low certainty in the available evidence [24], this decision was given a strong recommendation in the published guidelines [22]. Perhaps unsurprisingly, this recommendation represented the most controversial decision in the ASH ISTH NHF WFH guidelines and continues to divide opinion in the field [25,26]. In particular, concern has been expressed regarding how this decision may lead to the potential medicalization of a large number of people who have a combination of mild mucocutaneous bleeding (which has been reported in 23% of the general population) [27] and borderline plasma VWF levels (by definition, 2.5% of the general population have plasma VWF levels below the lower limit of the local normal range).

## 3 | IS LOW VWF A DISCRETE CLINIC-PATHOLOGICAL ENTITY?

The ASH ISTH NHF WFH guidelines specifically highlighted several important VWD research priorities, including the need for improved understanding of the heterogeneity underpinning quantitative VWD. Recent data from a combined analysis of the Low VWF Ireland Cohort (LoVIC) and Willebrand in the Netherlands (WiN) studies have provided some novel insights in this regard [28]. In line with previous studies, Atiq et al. [28] observed a progressive increase in plasma VWF:Ag levels with aging in many type 1 VWD (initial VWF levels < 30 IU/dL) patients. In approximately 30% of type 1 VWD patients, plasma VWF levels increased into the 30 to 50 IU/dL range over time (*WiN partially corrected*). Moreover, in a further 23% of type 1 VWD patients, plasma VWF:Ag levels increased into the normal range (>50 IU/dL) with progressive aging (*WiN normalized*). Importantly, the bleeding phenotype was similar in patients with VWF levels < 30 IU/dL, *WiN partially corrected*, and *WiN normalized*, suggesting that the age-related increases in VWF levels did not necessarily correct the bleeding phenotype of patients. The authors further showed that the age-dependent effect on VWF levels in the LoVIC cohort completely overlapped with the *WiN normalized* subgroup. Moreover, the Low VWF and *WiN normalized* cohorts were identical in terms of underlying pathophysiological mechanisms and the lower prevalence of pathological *VWF* sequence variants. Critically, the only significant difference between the LoVIC and *WiN normalized* groups was that the Low VWF cohort was first diagnosed at a significantly older age (33 years vs 25 years, respectively; *P* < .001) [28]. Together, these data clearly demonstrate that the first VWF testing in the clinic must, therefore, be understood as a snapshot taken at a single time point on a progressive age-dependent plasma VWF gradient.

## 4 | AGING AND TYPE 1 VWD DIAGNOSIS

These recent data have significant implications for clinical practice. For example, if we consider a female patient referred with mucocutaneous bleeding who undergoes initial VWF testing during childhood (eg, 10 years). Her plasma VWF:Ag level is 20 IU/dL, and the remainder of her laboratory testing is within the normal range. Consequently, she will be formally diagnosed with type 1 VWD. In contrast, if the same patient is not referred for formal VWF testing until she has reached 30 years of age, her plasma VWF level may have increased into the 30 to 50 IU/dL range, and so she will instead be labeled with a Low VWF diagnosis. Finally, if the same patient is not referred for initial VWF testing until she has reached 60 years of age, then her plasma VWF:Ag level may have increased >50 IU/dL, and consequently, she will be diagnosed as normal. Alternatively, if her bleeding history severity is impressive, she may instead be registered with a bleeding disorder of unknown cause diagnosis [11]. In summary, these recent data demonstrate that Low VWF does not constitute a distinct clinical or pathological entity. Rather, there is an age-dependent evolving phenotype in individuals with partial quantitative VWF deficiency, and Low VWF is a subgroup within heterogeneous type 1 VWD.

Collectively, the findings of Atiq et al. [28] support the ASH ISTH NHF WFH guidelines to remove the Low VWF subgroup classification. Nevertheless, it is important to emphasize that important clinical challenges posed by the marked heterogeneity within type 1 VWD have not gone away. Labeling this entire cohort with a type 1 VWD (rather than Low VWF) diagnosis may provide an important opportunity to reduce healthcare access barriers in the United States and decrease morbidity associated with delays to first diagnosis. However, in Europe, Australia, and other jurisdictions with universal healthcare provision models, the risk of further increasing the number of false-positive type 1 VWD diagnoses and overmedicalization may be of greater concern.

Additionally, the ASH ISTH NHF WFH panel’s decision to amend diagnostic terminology may have the potential to cause confusion among researchers in the field. For example, some recent studies have used the “Low VWF” terminology to refer to people with VWF levels of 30 to 50 IU/dL who do not have any bleeding phenotype. As previously highlighted in seminal publications from Evan Sadler [17,27,29], mild-to-moderate reductions in plasma VWF:Ag levels should be considered as a risk factor for bleeding rather than a constitutional bleeding disorder per se. Consistently, it is critical to emphasize that most individuals with plasma VWF levels in the 30 to 50 IU/dL range do not have any significant bleeding tendency. Consequently, healthy subjects with plasma VWF:Ag levels of 30 to 50 IU/dL without a significant bleeding phenotype should NOT be given a type 1 VWD diagnosis nor labeled “Low VWF,” irrespective of the reason for initial VWF measurement in these individuals. However, it is important to recognize that an individual with VWF levels in the 30 to 50 IU/dL range without a significant bleeding phenotype but who has not yet faced hemostatic challenges may have a higher risk of bleeding if there is a positive family history of VWD. This underscores the importance of assessing family history in the evaluation of patients with VWF levels in the 30 to 50 IU/dL range.

## 5 | CLINICAL APPROACH TO PARTIAL QUANTITATIVE VWD DIAGNOSIS

Following the introduction of the ASH ISTH NHF WFH guidelines [22], we suggest a diagnostic approach algorithm, as illustrated in the Figure. In line with the guideline recommendations, patients with VWF levels < 30 IU/dL (red) are diagnosed as type 1 VWD, and individuals with VWF levels > 50 IU/dL without any bleeding symptoms (green) are considered healthy. In the cohort of patients with VWF levels in the 30 to 50 IU/dL range, a careful review of bleeding history, family history of VWD, previous hemostatic challenges, and clinical gestalt is important. Patients with significant personal bleeding and VWF levels persistently in the 30 to 50 IU/dL range are registered with a type 1 VWD diagnosis [30]. In contrast, people with VWF levels of 30 to 50 IU/dL who have no bleeding history despite previous hemostatic challenges performed without any hemostatic prophylaxis are considered healthy and discharged from the clinic. In many ways, the most difficult subgroup to manage is those people who have plasma VWF levels in the 30 to 50 IU/dL range and have no or minimal bleeding history but have not yet been exposed to significant hemostatic challenges (yellow). Our practice is to label these individuals as “possible VWD” and to continue following them in the clinic. If they subsequently develop bleeding when subjected to hemostatic challenges in the future, then they are diagnosed with type 1 VWD. Conversely, if they experience no bleeding, with future challenges performed without any hemostatic prophylaxis, they are considered healthy and discharged. Based on our experience, most of these individuals will not bleed during invasive procedures, as they represent 2.5% of the general population who have borderline VWF levels by chance. If these individuals are treated prophylactically prior to future procedures, it will never be possible to determine whether they ever had a significant bleeding tendency in the first place. In addition, treating individuals with borderline VWF levels who lack a defined bleeding phenotype will lead to false-positive overdiagnosis of type 1 VWD in healthy individuals that may significantly impact their lives (eg, insurance, mortgage, or choice of career).

## 6 | CONCLUSIONS

Further clinical and basic scientific research will be needed to advance our understanding of the heterogeneity underlying partial quantitative VWD [7,15]. This is important as it has the potential to underpin global improvements in type 1 VWD diagnosis and management. To ensure that future research findings can be easily compared and extrapolated, the field would benefit if consensus could be reached regarding several specific issues. These include (i) what constitutes a bleeding challenge? (ii) how many bleeding challenges are sufficient to make a confident decision regarding bleeding phenotype? (iii) is “possible VWD” the preferred terminology for patients with VWF levels in the 30 to 50 IU/dL range who have not yet been exposed to hemostatic challenges, and (iv) should the “Low VWF” terminology be abandoned, or else specifically defined? Addressing these and other outstanding issues will be important in preventing inappropriate overdiagnosis of type 1 VWD while at the same time maximizing access to healthcare and minimizing diagnostic delays.

## Figures and Tables

**FIGURE:**
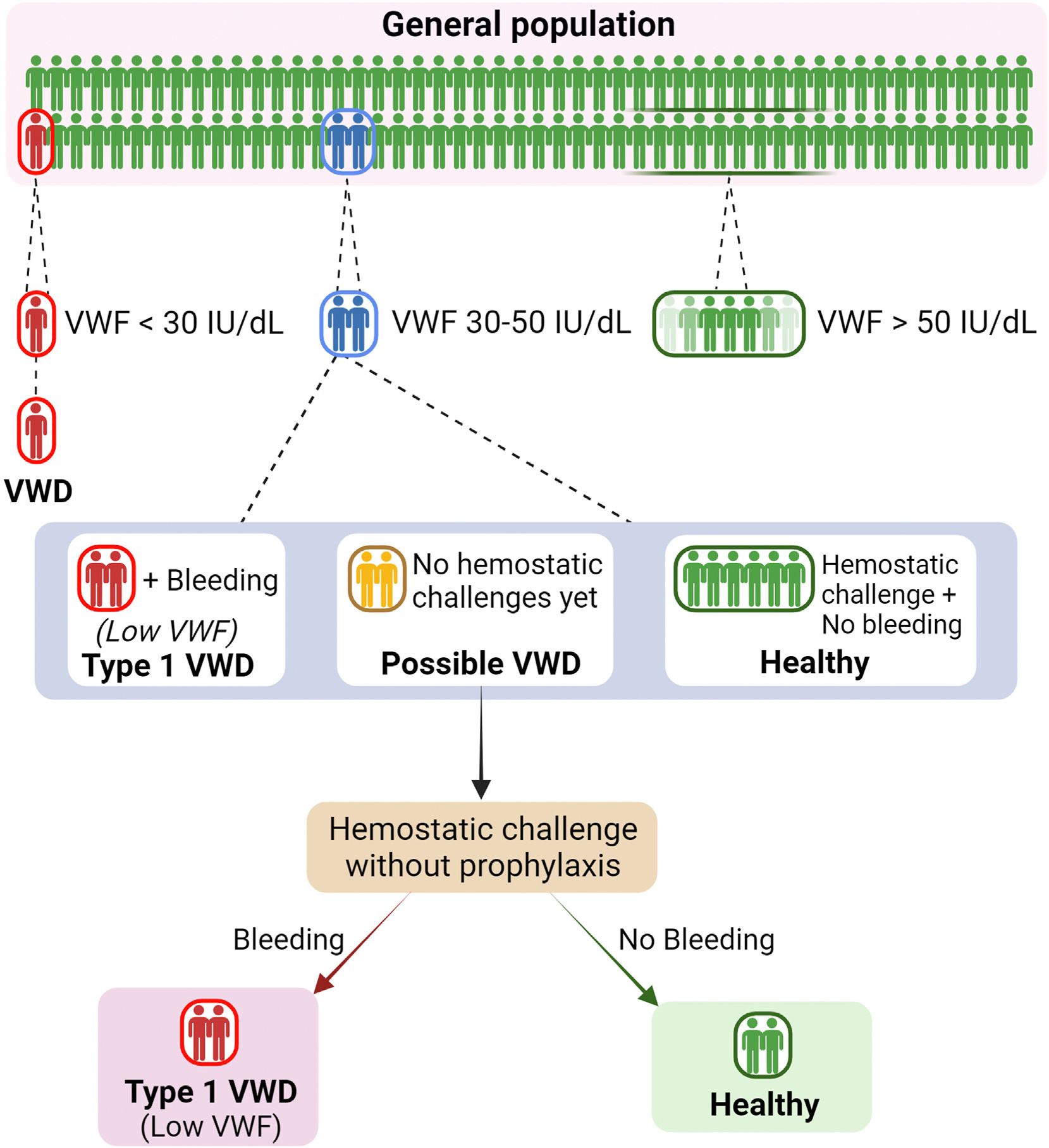
Proposed diagnostic approach algorithm for type 1 von Willebrand disease (VWD). By definition, 2.5% of the general population have plasma von Willebrand factor (VWF) antigen levels < 50 IU/dL. Individuals with VWF levels > 50 IU/dL are considered healthy (green). Those with VWF levels < 30 IU/dL are classified as type 1 VWD (red). Among individuals with VWF levels between 30 and 50 IU/dL, those with a significant bleeding phenotype are classified as type 1 VWD (red). In some jurisdictions, these patients may still be labeled with Low VWF. Individuals without bleeding, despite sufficient hemostatic challenges, are considered healthy and are discharged from the clinic (green). Individuals without a significant bleeding phenotype at present, who have not previously experienced hemostatic challenges, are categorized as “Possible VWD” (yellow). These patients are monitored until they experience hemostatic challenges. If they bleed after hemostatic challenges, they are diagnosed with type 1 VWD (red). If they do not bleed, they are classified as healthy and discharged from the clinic (green).
